# Nanoscale Structure of Langmuir–Blodgett Film of Bent-Core Molecules

**DOI:** 10.3390/nano12132285

**Published:** 2022-07-02

**Authors:** Fabrizio Corrado Adamo, Federica Ciuchi, Maria Penelope De Santo, Paola Astolfi, Isabelle Warner, Eric Scharrer, Michela Pisani, Francesco Vita, Oriano Francescangeli

**Affiliations:** 1 SIMAU Department, Polytechnic University of Marche, Via Brecce Bianche, 60131 Ancona, Italy; f.c.adamo@staff.univpm.it (F.C.A.); p.astolfi@univpm.it (P.A.); m.pisani@univpm.it (M.P.); 2 CNR-Nanotec c/o Physics Department, University of Calabria, Ponte Bucci, Cubo 31C, 87036 Arcavacata di Rende, Italy; federica.ciuchi@cnr.it (F.C.); maria.desanto@fis.unical.it (M.P.D.S.); 3 Department of Chemistry, University of Puget Sound, Tacoma, WA 98416, USA; iwarner@alumni.pugetsound.edu (I.W.); escharrer@pugetsound.edu (E.S.)

**Keywords:** bent-core mesogens, Langmuir–Blodgett films, X-ray reflectivity

## Abstract

Bent-core mesogens (BCMs) are a class of thermotropic liquid crystals featuring several unconventional properties. However, the interpretation and technological exploitation of their unique behavior have been hampered by the difficulty of controlling their anchoring at surfaces. To tackle this issue, we report the nanoscale structural characterization of BCM films prepared using the Langmuir–Blodgett technique. Even though BCMs are quite different from typical amphiphilic molecules, we demonstrate that stable molecular films form over water, which can then be transferred onto silicon substrates. The combination of Brewster angle microscopy, atomic force microscopy, and X-ray reflectivity measurements shows that the molecules, once transferred onto a solid substrate, form a bilayer structure with a bottom layer of flat molecules and an upper layer of upright molecules. These results suggest that Langmuir–Blodgett films of BCMs can provide a useful means to control the alignment of this class of liquid crystals.

## 1. Introduction

Since their discovery [1], bent-core mesogens (BCMs) have opened new horizons in the science of thermotropic liquid crystals (LCs). They form several peculiar phases, known as banana phases, attracting considerable interest because of their unique polar and chiral properties [2,3,4]. More recently, scientific research has focused on the unconventional behavior of their nematic (N) phase [5,6,7], suggestive of the long-sought-after nematic biaxiality [8,9,10] and ferroelectricity [11,12]. The origin of these unique features is the presence, throughout the N temperature range, of nanosized clusters of layered molecules, known as cybotactic groups, exhibiting a biaxial and possibly polar order [13,14].

Despite this considerable interest, both the fundamental study and technological application of BCMs have been hampered by a lack of reliable means to control their surface alignment. Well-known surface treatments, which allow for the fine control of the director alignment in conventional (calamitic) nematics, have provided unpredictable results when used with BCMs, often leading to questionable interpretations of the experimental data [15,16,17]. For instance, the supposed biaxiality of some nematic BCMs, deduced from the in-plane optical anisotropy of homeotropically aligned samples, has been reinterpreted in terms of the anchoring transition of the uniaxial N director to a tilted configuration [18,19,20]. A deeper understanding of the BCM anchoring properties is thus a critical issue for the development of the promising field of LC science.

In this context, the deposition of the Langmuir films of BCMs on proper substrates can provide both an ideal system for the study of LC anchoring on the molecular scale and a suitable approach to obtain aligning surfaces with controlled anchoring conditions [21,22,23,24,25,26,27,28,29,30,31,32,33]. A Langmuir monolayer is formed at the air–water interface by dispersing insoluble molecules over water; compression of the film by moving mechanical barriers determines the molecular arrangement in the monolayer, which can then be transferred onto a solid substrate. The stability of a Langmuir film and the orientation of the molecules on the water critically depend on the interplay between the molecular hydrophobic and hydrophilic moieties. Langmuir films are conventionally prepared with amphiphilic molecules. In contrast, typical BCMs have two hydrophobic alkyl tails of variable length and a more hydrophilic aromatic core. Thus, obtaining an upright orientation of the molecules over water, and then over a solid substrate, is much easier if one of the tails is modified by the insertion of a hydrophilic end group. This approach has been discussed in several papers, and the structural features of the resulting films are generally well understood [22,28,29,30,33]. Additionally, the possibility of obtaining stable thin films from conventional BCMs with hydrophobic aliphatic tails has been reported in the literature, with tentative structural models deduced from qualitative considerations of the molecular hydrophobic/hydrophilic properties, and from geometrical arguments about the area per molecule in the isotherm. The first of these reports is ambiguous about the molecular organization [21]: despite the authors conjecturing a multilayer structure over the water (because of the small co-area obtained by extrapolating the compression isotherm to the baseline), they assumed a monolayer structure for the Langmuir–Blodgett deposition on the solid substrate. Based on similar considerations of the co-area, combined with Brewster microscopy and surface potential measurements, Zou et al. proposed a double-layer model for their Langmuir films; although they could not resolve the exact film structure, they hypothesized a first layer with the core in contact with the surface and overlying layers with more upright molecules [22]. Subsequently, a similar structure has been assumed in other papers [23,24,25,26]. In [25,26], this structural model was supported by thickness measurements: applying interference microscopy and capacitance measurements to multilayer films made by different numbers of Langmuir–Schaefer depositions, the thickness of a single deposition was extrapolated; this value was compatible either with the expected thickness of monolayers with upright molecules or with that of a more complicated bilayer structure, with a top layer of slightly tilted upright molecules standing over an underlying layer of horizontally aligned molecules. The propensity of BCMs to lie parallel to the water surface was confirmed by single-molecule molecular dynamics simulations [34]; similar studies performed on pairs of molecules indicated the role of the water surface in the favoring of the molecular packing and the formation of stable complexes [35]. Although the above-mentioned papers provide useful clues on the possible structure of these unconventional Langmuir films, the proposed models are only qualitative and lack direct experimental verification.

**Figure 1:**
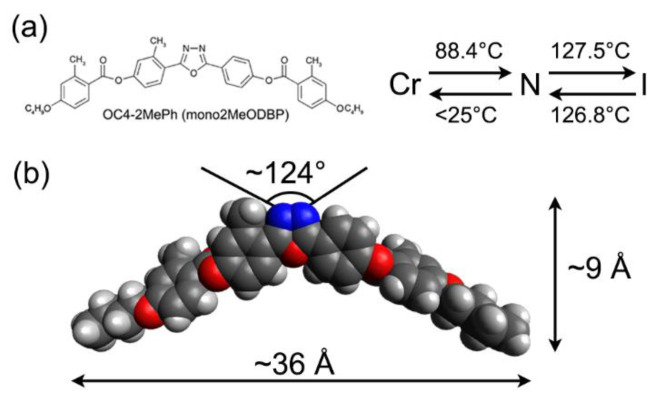
(**a**) Chemical structure and phase diagram of OC4-2MePh(mono2MeODBP) upon heating and cooling; (**b**) 3D model of the molecule with typical dimensions and bending angle.

To clarify this issue, we present in this paper a structural study of Langmuir–Blodgett BCM films based on X-ray reflectivity (XRR) measurements. The results were supplemented by atomic force microscopy (AFM) and Brewster angle microscope (BAM) experiments, the latter performed on Langmuir films over water. The BCM chosen for film preparation, a compound known as OC4-2MePh(mono2MeODBP), features short butoxy terminal chains attached to a laterally substituted oxadiazole bisphenol core. Its molecular structure, phase diagram, and optimized geometry are shown in Figure 1. This compound belongs to a family of laterally substituted oxadiazole-based bent-core nematics, which has been extensively studied in the past to highlight the effects of lateral substituents on the compounds’ phase diagram and structural properties [9,20,36,37,38,39]. In particular, the three lateral methyl groups impart a number of peculiar features to OC4-2MePh(mono2MeODBP): (i) they considerably shift the nematic temperature range toward lower temperatures compared with those of the unsubstituted compound; (ii) they allow the supercooling of the N phase down to room temperature in a highly viscous metastable state; (iii) they are responsible for the local biaxial arrangement of the molecules in the N phase, as evidenced by X-ray diffraction data [9,39].

## 2. Materials and Methods

The synthesis and mesogenic behavior of OC4-2MePh(mono2MeODBP) (Figure 1) were detailed elsewhere [36]. Its bulk structural properties in the N phase have been thoroughly studied by means of X-ray diffraction experiments [9,36,37].

Thin films over water were prepared using NIMA Langmuir troughs (NIMA Technology Ltd., Coventry, England) available at the laboratories of the University of Calabria and at the Partnership of Soft Condensed Matter (PSCM) of the European Synchrotron Radiation Facility (ESRF), Grenoble (France). The film formation was monitored in real time by an Accurion EP3 Brewster angle microscope (Accurion GmbH, Goettingen, Germany). This microscope uses a *p*-polarized laser beam (*λ* = 532 nm) to illuminate the liquid surface at an incidence angle matching the Brewster angle of the water–air interface. Because in this condition the reflectance from a pure water–air interface is null, the entire signal viewed in the microscope CCD camera is produced by the reflectance of the Langmuir layer.

The trough was filled with pure water from an ELGA PURELAB Flex water purification system (with a resistivity of 18.2 MΩ/cm) (ELGA LabWater, Wycombe, UK). The BCM was dissolved in chloroform at 0.1 mg/mL and spread over the water surface to an initial area per molecule of *A* ≈ 130 Å^2^. After the equilibration and evaporation of the solvent, the barriers were moved at a constant rate of 70 cm^2^/min, and the corresponding compression/expansion isotherms (surface pressure Π vs. area per molecule *A*) were recorded.

The films were deposited onto silicon substrates ((100)-cut *n*-doped, previously treated with piranha solution) using the Langmuir–Blodgett technique: the substrate was vertically immersed into the water before spreading the BCM solution; hence, the solution was spread and the barriers were closed until the desired deposition pressure was reached. Lastly, a motor extracted the substrate from the water with the BCM film adhering to the silicon surface. The deposition pressure was Π = 12 mN/m, corresponding to an area per molecule of *A* ≈ 40 Å^2^. After the deposition, the excess water was left to evaporate before performing any characterizations of the films.

AFM (Multimode 8 with a Nanoscope V controller, Bruker, Santa Barbara, CA, USA) was used to investigate the film morphology and uniformity. Data were acquired in tapping mode using silicon cantilevers (model TAP150, Bruker, Santa Barbara, USA).

XRR was used to determine the density profile normal to the film surface, the layer thickness, and correlated roughness. The XRR experiments were performed at the ID03 beamline of the ESRF, Grenoble (France). The samples were placed on a high-precision hexapod support, while a photon-counting detector MAXIPIX (ESRF, Grenoble, France) was mounted on a *z*-axis vertical diffractometer. The beam width and height were 200 μm and 40 μm, respectively, while the beam wavelength was *λ* = 0.55 Å. The measurements were performed at room temperature. The reflectivity curves were analyzed with the open source software GenX 3.2 [40], which uses the Parratt algorithm to simulate specular reflectivity from multilayer samples and fit the experimental data [41]. The roughness of each layer was modeled with a Gaussian distribution per the Nevot–Croce theory [42,43]. For very low incidence angles, when the footprint of the incoming beam may exceed the sample size, GenX is able to correct the reflected intensity by considering the values of the beam width and sample length. In modeling the sample, it was important to consider the few-nanometers-thick layer of native silicon oxide (SiO_2_) that is typically present on the surface of single-crystal silicon substrates [44]. The silicon substrate parameters were kept fixed at values known from the literature, whereas only the electron density was kept fixed for the overlying SiO_2_ layer. The BCM film was modeled as a stack of four layers, as described in the following section.

## 3. Results and Discussion

**Figure 2:**
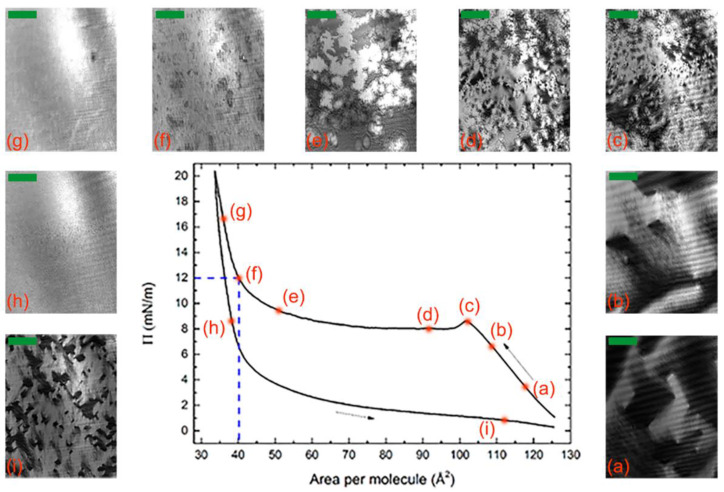
(**a**–**i**) Isotherm of a complete compression (leftward arrow) and expansion (rightward arrow) cycle and corresponding sequence of BAM images (the scale bar in each image corresponds to 100 μm). The compression/expansion rate was 70 cm^2^/min. The dashed lines indicate the deposition parameters.

A typical Π-*A* isotherm taken during a full compression/expansion cycle of the BCM Langmuir film is shown in Figure 2 together with a sequence of BAM images. After spreading, the decrease in energy associated with molecular aggregation overcame the entropy loss, and the molecules started to form floating islands, freely moving over the water surface (Figure 2a). Within these islands, it can reasonably be assumed that the molecules lay flat on the water surface, with the hydrophilic carbonyl oxygens of the mesogen core close to the water and the hydrophobic end-chains elevated [34]. This hypothesis is supported by the relatively large area per molecule value.

When the barriers started to compress, the islands drew near to one another then coalesced (Figure 2b); consequently, the pressure increased until the water surface was almost completely covered. Hence, further compression induced a reorganization of the film, as indicated by the bump in the isotherm at *A* ≈ 102 Å^2^/molecule (Figure 2c). Subsequently, the isotherm showed a plateau where the area per molecule decreased but the pressure remained approximately constant; this behavior is associated to the reorganization of the molecules into a new configuration (Figure 2d,e). In our study, this reasonably corresponded to the formation of a double layer. This is particularly evident at the end of the plateau region (Figure 2e), where the BAM image showed the coexistence of regions of different brightness. As the brightness in BAM images is proportional to the square of the thickness of the Langmuir film [45], this indicates the concurrence of regions of different thickness. At *A* ≈ 40 Å^2^/molecule (Figure 2f), the BAM image became more homogeneous, and an abrupt increase in the isotherm slope simultaneously manifested, indicating a phase transition into a solid-like arrangement of the molecules with an almost uniform thickness across the film. At a higher pressure (Figure 2g), the molecules formed a tightly packed film that remained equally compact after the expansion of the barriers (Figure 2h), thus suggesting a strong interaction among the molecules. Upon further expansion, the molecules remained relatively well packed even at a higher value of the area per molecule (Figure 2i).

The hysteresis observed during the compression/expansion cycle—a quite common feature of Langmuir films—reflects differences between the aggregation (upon compression) and the relaxation (upon expansion) processes, e.g., their different kinetics. The area between the curves provides the free energy that remains stored in the film at the end of the cycle, e.g., in the form of interaction energy among the molecules. The measured value of 738 cal/mol is relatively large if compared with data reported in the literature for other systems (e.g., 300 cal/mol for mixed monolayers of fatty acids [46] and 626 cal/mol for myelin monolayers [47]), which indicates a strong cohesive intermolecular energy.

**Figure 3:**
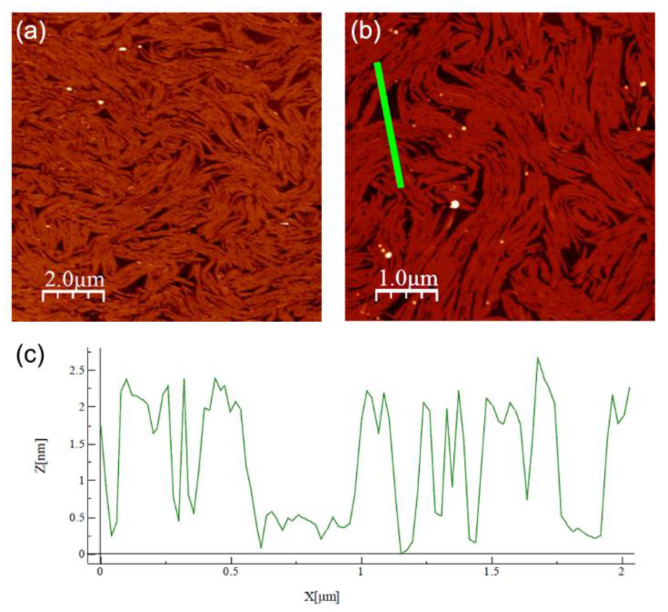
(**a**,**b**) AFM images of Langmuir–Blodgett films deposited on silicon at Π = 12 mN/m, taken at different positions and magnifications. (**c**) Thickness profile measured along the green bar in (**b**).

We chose point (f) of the isotherm in Figure 2 (Π = 12 mN/m and *A* = 40 Å^2^/molecule) as the set point for the Langmuir–Blodgett deposition of the film on a silicon substrate. After evaporation of the excess water, the films were analyzed with AFM. Representative images taken at different magnifications are shown in Figure 3a,b. The film covered nearly all the substrate with a thickness modulation of ~22 Å, as indicated by the depth profile shown in Figure 3c. However, the film was not uniform as it exhibited a supramolecular fibrous structure, with each fiber having a width in the order of 10^2^ nm and a length in the order of 1 μm. The fibers tended to lie parallel to one another, forming well-aligned domains, the size of which was in the order of a few square micrometers. The fiber alignment was lost over larger distances and no preferential orientation was identifiable on the sample surface. Such behavior resembles that of the N director **n** in a conventional LC sample with tangential alignment (i.e., with the director lying in the plane of the substrate without any in-plane preferential orientation). The XRR measurements discussed below indicate that the deposited films consisted of two molecular layers, with the molecules of the underlying layer lying parallel to the substrate. Hence, we hypothesize that the fibrous structure observed with AFM reflects the local alignment induced in the film by those molecules.

**Figure 4:**
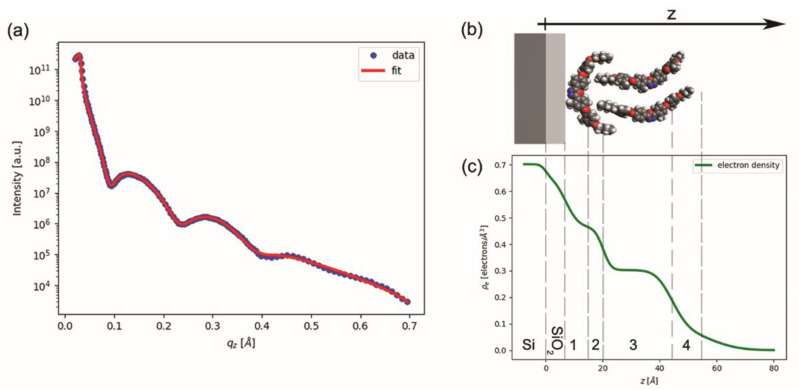
(**a**) XRR curve: experimental data (blue dots) and fitting curve (red line). (**b**) Structural model of the BCM film over the silicon and silicon oxide layers (grey and light grey, respectively). (**c**) Electron density (*ρ_e_*) vs. film thickness (*z*), as obtained from fitting the XRR curve with the multilayer model described in the text. Grey dashed lines show the resulting thickness of each layer (see Table 1).

The films were then characterized using XRR, obtaining the typical reflectivity curve shown in Figure 4a (the blue dots). Attempts to fit the data by modeling the BCM film as a single layer over the silicon substrate (with the addition of an intermediate thin layer of native silicon oxide) proved unsuccessful. We found that a minimum of four distinct layers was necessary to model the BCM film and properly fit the experimental data. If we allowed the fit parameters (the electron density, thickness, and roughness of each BCM layer, plus the thickness and roughness of the underlying SiO_2_ layer) to vary independently without constraints, then we obtained a total BCM thickness of 44.2 Å and a BCM electron density profile (not shown) monotonically decreasing with the *z* coordinate (*z* being the distance from the bottom of the film, as defined in Figure 4b). Although this fit reproduced the experimental data well, we could attribute no physical meaning to the BCM layers, as they were merely a mathematical artifice to model the actual electron density profile of the BCM film. However, considering that the molecular length of our BCM is expected to be ~36 Å, this preliminary fit clearly indicated that our film could not be constituted by a single molecular layer.

Based on these preliminary results, we performed a refined analysis of the XRR data, linking the multilayer structure used for fitting with a physical model of the BCM film. By considering (*i*) the hydrophobicity of the BCM tails, incompatible with water and hydrophilic SiO_2_; (*ii*) the BAM images and the Π-*A* isotherm recorded during the compression of the Langmuir films, indicating a multilayer arrangement of the molecules over water; (*iii*) the XRR indication of a larger electron density in the proximity of the substrate and the estimated thickness of the BCM film; and (*iv*) the qualitative models reported in the literature for similar BCM films [22,23,24,25,26], we hypothesized the film structure sketched in Figure 4b: a compact (solid-like) bottom layer of molecules lying down over the substrate with their tails pointed upward, and a looser upper layer of upright (or slightly tilted) molecules. To confirm this picture, we modeled the BCM film as a sequence of four layers (not necessarily coinciding with those used for the preliminary fit mentioned above), containing, from the bottom to the top: the flat aromatic cores of the underlying molecules (Section 1); both tails of the underlying molecules and the lower tails of the overlying molecules (Section 2); the upright cores of the overlying molecules (Section 3); and the upper tails of the overlying molecule (Section 4).

The electron density *ρ_e,i_* of the *i*th section was written as:

(1) $$\rho_{e,i}=\frac{N_{i}}{d_{i}\;S\;}$$

with *S* being the film area associated to each molecule in the bottom layer, *d_i_* being the thickness of each section, and *N_i_* being the number of electrons in the volume *V_i_ = Sd_i_*. Knowing the BCM chemical structure, we expressed *N_i_* in terms of the number *n* of overlying molecules for each underlying molecule (i.e., the ratio between the number of molecules in the top layer and the number of molecules in the bottom layer), as follows: for layer 1, *N*_1_ *= Z_C_* (*Z_C_ =* 262 is the number of electrons in the mesogen core); layer 2 contained two tails from the underlying molecule and *n* tails from the overlying molecules, so that *N*_2_ *= (n +* 2) *Z_T_* (*Z_T_* = 41 is the number of electrons in one butoxy tail); for layer 3, *N*_3_ *= nZ_C_*; and for layer 4, *N*_4_ *= nZ_T_*. In this way, we could relate the electron density of each layer to the molecular structure and to the physically meaningful parameters *S*, *n*, and *d_i_*. This model was then used to fit the XRR data, with *S*, *n*, *d_i_*, and the roughness *σ_i_* of each BCM section (plus the thickness and roughness of the underlying SiO_2_ layer) as the fitting parameters. The resulting best fit to the experimental data is shown in Figure 4a: the computed best fit (the red curve) well-matched the experimental data (the blue points). The corresponding best fit parameters were *S* = 69.1 Å^2^ and *n* = 1.92, whereas the best fit values of thickness and roughness are reported in Table 1; this table also shows the computed electron density of each section.

To describe the electron density as a stepwise function of *z* would be a strong simplification; instead, we consider the relatively large roughness values to be a mathematical means to model the smoothing effect of molecular flexibility and positional and orientational disorder. When the roughness is considered, one obtains the more realistic density profile shown in Figure 4c, which can be directly compared with the molecular model shown in Figure 4b. However, the image in Figure 4b is simply a 3D graphical representation of the average molecular arrangement and was not obtained by full molecular dynamics simulations. In particular, whereas the molecular cores are expected to be quite rigid and thermodynamically stable, traits such as the configuration of the terminal tails and the position of the upper molecules with respect to the lower ones may significantly change, also within the same film.

The most evident feature in the profile shown in Figure 4c is the presence of two regions of approximately constant density—a short high-density region close to the substrate and a much larger low-density region centered at *z* ≈ 30 Å—which correlated well with the expected positions of the BCM cores in the bottom and top layer, respectively, as shown in the model of Figure 4b. Beyond *z* ≈ 40 Å, the electron density very slowly decreases to zero, suggesting a significant level of disorder in the upper molecular layer (and in particular in the BCM tails at the film–air interface). Overall, the thickness values provided by the fit were very reasonable: the two bottom sections of the BCM film (Section 1 and Section 2) had a combined thickness of about 13 Å, which compares well with the expected thickness of a BCM molecule lying flat with its terminal tails pointed upward, as shown in Figure 4b. The thickness of Section 3 was in reasonable agreement with the thickness of the BCM aromatic core (~22 Å); by contrast, the combined thickness of Section 2, Section 3 and Section 4 (41 Å) was slightly larger than the expected molecular length (~36 Å). This discrepancy is mainly due to the thickness of Section 4, which was significantly larger than the expected length of butoxy terminal tails. However, the level of roughness of Section 4 was significant and comparable to its thickness; this added uncertainty to any attempt to establish a well-defined film thickness. These results suggest large fluctuations in the thickness of the upper layer, in agreement with the results of the AFM measurements.

Likewise, the value obtained for the base area *S* of a flat molecule was reasonable, indicating a compact packing of the aromatic cores with lifted end tails. By contrast, the value of *n* ≈ 2 upright molecules for each underlying molecule suggests an overlying layer that is much less dense and hence much less ordered. This can explain the discrepancy between the total film thickness measured with XRR and the value provided by AFM: by assuming the substrate was covered by a tightly packed layer of flat molecules, it can be conjectured that the morphology shown with the AFM scans reflects the fluctuations/undulations of the more inhomogeneous and less compact overlying layer, which is also suggested by the significant roughness (in the order of ±5 Å) present in the baseline of the AFM thickness profile shown in Figure 3c.

## 4. Conclusions

In this study, we demonstrated the formation of stable Langmuir films of BCM molecules over water and the possibility of transferring them onto solid substrates via Langmuir–Blodgett deposition. XRR measurements, combined with complementary techniques, provided robust evidence of a double layer organization in the transferred films: a bottom layer of tightly packed flat molecules and a looser upper layer of upright molecules (in a number ratio of about 1:2). Although this structure has been conjectured in the past, our results represent the first direct proof of this unconventional arrangement. Regardless of the details of the fit, the relatively large film thickness and the electron density monotonically decreasing with *z* both exclude alternative structural models for the BCM film, e.g., a monolayer of upright or flat molecules.

Furthermore, these findings demonstrate the suitability of Langmuir-based techniques to prepare thin films of liquid crystalline materials, even when their molecular structure is substantially different from that of typical amphiphilic molecules. We expect that this approach can allow for the fine control of the anchoring conditions (both in-plane and out-of-plane) in bent-core liquid crystals that otherwise cannot be easily aligned by conventional means.

## Figures and Tables

**Table 1:** Thickness *d*, electron density *ρ_e_*, and roughness *σ* of the layer stack used to model the sample: a Si substrate, an intermediate layer of native SiO_2_, and four sections for the BCM film. The thickness and roughness values were provided by the fitting procedure together with the values *S* = 69.1 Å^2^ and *n* = 1.92. The electron density of each BCM layer was calculated from these values using the model described in the text. The Si substrate parameters and the electron density of SiO_2_ were taken from the literature and kept fixed.

	*d* (Å)	*ρ_e_* (Å^−3^)	*σ* (Å)
Si	∞	0.70	1.5
SiO_2_	7.0	0.66	3.6
BCM 1	8.1	0.47	1.5
BCM 2	5.0	0.47	2.2
BCM 3	24.1	0.30	4.5
BCM 4	11.8	0.10	8.9

## Data Availability

The data presented in this study are available upon request from the corresponding author.
